# 14-3-3 Regulates Actin Filament Formation in the Deep-Branching Eukaryote *Giardia lamblia*

**DOI:** 10.1128/mSphere.00248-17

**Published:** 2017-09-13

**Authors:** Jana Krtková, Jennifer Xu, Marco Lalle, Melissa Steele-Ogus, Germain C. M. Alas, David Sept, Alexander R. Paredez

**Affiliations:** a Department of Biology, University of Washington, Seattle, Washington, USA; b Department of Experimental Plant Biology, Faculty of Science, Charles University, Prague, Czech Republic; c Department of Infectious Diseases, Istituto Superiore di Sanità, Rome, Italy; d Department of Biomedical Engineering and Center for Computational Medicine and Bioinformatics, University of Michigan, Ann Arbor, Michigan, USA; University at Buffalo

**Keywords:** 14-3-3, evolutionary cell biology, Rho GTPase, actin

## Abstract

The phosphoserine/phosphothreonine-binding protein 14-3-3 is known to regulate actin; this function has been previously attributed to sequestration of phosphorylated cofilin. 14-3-3 was identified as an actin-associated protein in the deep-branching eukaryote *Giardia lamblia*; however, *Giardia* lacks cofilin and all other canonical actin-binding proteins (ABPs). Thus, the role of *G. lamblia* 14-3-3 (Gl*-*14-3-3) in actin regulation was unknown. Gl-14-3-3 depletion resulted in an overall disruption of actin organization characterized by ectopically distributed short actin filaments. Using phosphatase and kinase inhibitors, we demonstrated that actin phosphorylation correlated with destabilization of the actin network and increased complex formation with 14-3-3, while blocking actin phosphorylation stabilized actin filaments and attenuated complex formation. *Giardia*’s sole Rho family GTPase, Gl-Rac, modulates Gl-14-3-3’s association with actin, providing the first connection between Gl-Rac and the actin cytoskeleton in *Giardia*. *Giardia* actin (Gl-actin) contains two putative 14-3-3 binding motifs, one of which (S330) is conserved in mammalian actin. Mutation of these sites reduced, but did not completely disrupt, the association with 14-3-3. Native gels and overlay assays indicate that intermediate proteins are required to support complex formation between 14-3-3 and actin. Overall, our results support a role for 14-3-3 as a regulator of actin; however, the presence of multiple 14-3-3–actin complexes suggests a more complex regulatory relationship than might be expected for a minimalistic parasite.

**IMPORTANCE** *Giardia* lacks canonical actin-binding proteins. Gl-14-3-3 was identified as an actin interactor, but the significance of this interaction was unknown. Loss of Gl-14-3-3 results in ectopic short actin filaments, indicating that Gl-14-3-3 is an important regulator of the actin cytoskeleton in *Giardia*. Drug studies indicate that Gl-14-3-3 complex formation is in part phospho-regulated. We demonstrate that complex formation is downstream of *Giardia*’s sole Rho family GTPase, Gl-Rac. This result provides the first mechanistic connection between Gl-Rac and Gl-actin in *Giardia*. Native gels and overlay assays indicate intermediate proteins are required to support the interaction between Gl-14-3-3 and Gl-actin, suggesting that Gl-14-3-3 is regulating multiple Gl-actin complexes.

## Abstract (precis)

*Giardia* lacks canonical actin-binding proteins. Gl-14-3-3 was identified as an actin interactor, but the significance of this interaction was unknown. Loss of Gl-14-3-3 results in ectopic short actin filaments, indicating that Gl-14-3-3 is an important regulator of the actin cytoskeleton in *Giardia*. Drug studies indicate that Gl-14-3-3 complex formation is in part phospho-regulated. We demonstrate that complex formation is downstream of *Giardia*’s sole Rho family GTPase, Gl-Rac. This result provides the first mechanistic connection between Gl-Rac and Gl-actin in *Giardia*. Native gels and overlay assays indicate intermediate proteins are required to support the interaction between Gl-14-3-3 and Gl-actin, suggesting that Gl-14-3-3 is regulating multiple Gl-actin complexes.

## INTRODUCTION

The protein 14-3-3 belongs to a family of highly conserved eukaryotic proteins whose role is to regulate target proteins through binding of specific phosphoserine/phosphothreonine motifs. Through recognition and binding of these specific motifs, 14-3-3 functions in a variety of cellular processes, including cytoskeletal regulation. 14-3-3 can act as an adapter in order to activate/inhibit protein function, change intracellular localization of bound cargos, or mediate formation of multiprotein complexes (1–8). In the current model for higher eukaryotes, 14-3-3 regulates actin through phospho-dependent sequestration of the actin-depolymerizing protein cofilin (4, 9). The existence of multiple 14-3-3 isoforms in higher eukaryotes complicates the relationship between 14-3-3 and actin, leading to discrepant results about whether 14-3-3 directly interacts with actin (2). Consistent with additional interaction/regulatory mechanisms, actin has been identified as an interactor of 14-3-3 in plant and animal 14-3-3 proteomic data sets where cofilin was not found (10–12). Indeed, 14-3-3σ was recently reported to be upregulated in breast cancer cells, where it forms a complex with actin and intermediate filament proteins that is utilized for cell motility during breast tumor invasion (13). The complex was also found to play a role in actin sequestration, as depletion of 14-3-3σ led to an increase in filamentous actin. Whether complex formation between monomeric actin and 14-3-3 is a broadly utilized mechanism of actin regulation remains an open question.

*Giardia lamblia* (synonymous with *G. intestinalis* and *G. duodenalis*), is a protozoan parasite that belongs to a deep-branching group of eukaryotes known as *Excavata*. *Giardia*, in concordance with its phylogenetic position, has an evolutionarily divergent actin with only 58% average identity to other actin homologs and lacks the canonical actin-binding proteins (ABPs) once thought common to all eukaryotes (Arp2/3 complex, formin, wave, myosin, cofilin, etc.) (14–16). Other excavates, such as *Trichomonas vaginalis* and *Spironucleus salmonicida*, also lack many canonical actin-binding proteins, suggesting that the core actin regulators conserved in plants, animals, and fungi may not have solidified their cellular roles before the ancestors of these excavates branched from the eukaryotic tree (17–20). The presence of ABPs in the closely related diplomonad *Spironucleus*, not found in *Giardia* (20), suggests that *Giardia*’s minimalism is in part due to reductive evolution. Nevertheless, *Giardia* actin (Gl-actin) functions in conserved cellular processes, including membrane trafficking, cytokinesis, polarity, and control of cellular morphology (21). The mechanism for actin recruitment and regulation for these processes remains poorly understood. The only conserved actin regulator identified in *Giardia* is a Rho family GTPase, Gl-Rac, which can promote changes in actin organization without any of the actin-binding proteins known to link small G-protein signaling to the actin cytoskeleton (21). Notably, 14-3-3 has been shown to integrate G-protein signaling to the actin and tubulin cytoskeleton in *Dictyostelium discoideum* (7); thus, it potentially links Gl-Rac to the actin cytoskeleton in *Giardia*. Through actin affinity chromatography and MudPIT analysis, the single 14-3-3 homolog (Gl-14-3-3) of *Giardia* was identified as an actin-associated protein (19). Likewise, actin has been identified as part of the 14-3-3 interactome in *Giardia* (22). Here we set out to address whether Gl-14-3-3 has a role in regulating the Gl-actin cytoskeleton, characterize the nature of the interaction, and determine if *Giardia*’s sole Rho family GTPase, Gl-Rac, is upstream of this association.

## RESULTS

**FIG 1:**
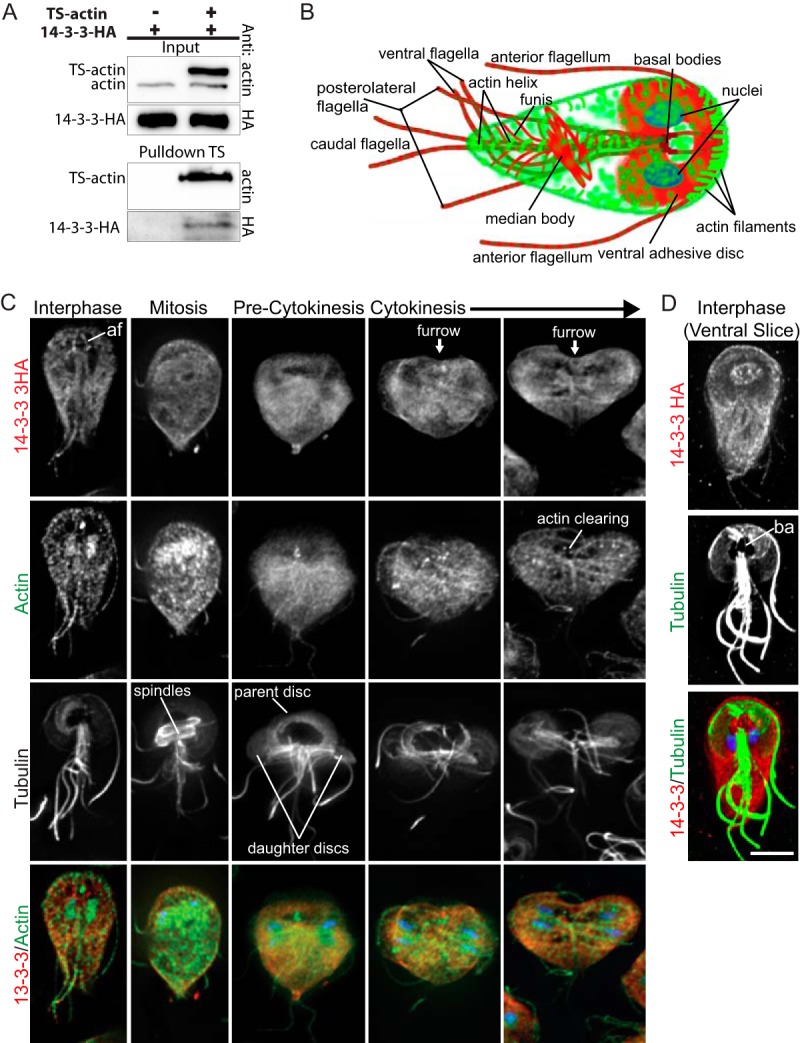
14-3-3 is associated with monomeric actin. (A) Pulldown of TS-actin demonstrating that 14-3-3 interacts with monomeric actin. (B) Diagram of actin (green) and tubulin (red) cytoskeletal structures found in interphase *Giardia* trophozoites. (C) Gl-14-3-3–HA (red), Gl-actin (green), tubulin (grayscale), and DNA (blue) localized in interphase, mitosis, and cytokinesis. Gl-14-3-3–HA was enriched along the intracytoplasmic portion of the anterior flagella (af). (D) Gl-14-3-3–HA (red), tubulin (green), and DNA (blue) projection spanning the ventral region only. Note Gl-14-3-3–HA in the microtubule bare area (ba) of the ventral disc (conduit for membrane trafficking). Scale bar, 5µm.

We previously reported that Gl-14-3-3 complexes with Gl-actin, and the biochemical conditions used to isolate interactors suggested the interaction was likely with monomeric G-actin (19). Pulldown of “bait” (Twin-Strep-tag)-tagged Gl-actin (TS-actin) expressed over endogenous actin supports this assertion. Cleared lysates (Fig. 1A) contain both endogenous Gl-actin and TS-actin. If Gl-14-3-3 bound to F-actin or complexes containing dimeric actin, then the filaments would contain a mixture of native and tagged actin. After pulldown of TS-actin with StrepTactin resin in buffers not expected to support filament formation, only TS-actin was detected (Fig. 1A). This finding is consistent with the Gl-14-3-3 complex containing monomeric actin, but does not exclude interaction with F-actin.

Next we questioned whether there is sufficient Gl-14-3-3 to act as a major actin regulator. Gl-actin and Gl-14-3-3 levels have not been measured in *Giardia*. For actin in particular, assignment of the intracellular concentration relative to the critical concentration has important regulatory implications. If the concentration of actin is above the critical concentration, then *Giardia* would require a mechanism to sequester actin. Using purified proteins as standards and custom antibodies to Gl-actin and Gl-14-3-3, we measured actin and 14-3-3 concentrations in *Giardia* trophozoite extracts. We found that 10 µg of extract contained 102.5 ± 7.4 ng of Gl-14-3-3 and 70.7 ± 16.4 ng of Gl-actin or ~1.8 pmol of 14-3-3 dimer and ~1.7 pmol of actin (see Fig. S1 in the supplemental material). Our measurement of actin at 70 ng per 10 µg of total cellular extract can be extrapolated to ~4.7 µM actin (where 16,927 cells = 10 µg and 1 cell = 199.8 µm^3^ [23]). Compared with other eukaryotes, this actin concentration is relatively low, yet the value is at least 5× higher than the concentration needed to form filaments (21), indicating that some level of actin sequestration is likely needed to prevent spontaneous filament formation. Since Gl-14-3-3 associates with monomeric actin, a portion of the total actin pool, there appears to be sufficient Gl-14-3-3 to bind and modulate actin as well as regulate the many other Gl-14-3-3 target proteins.

10.1128/mSphere.00248-17.1 FIG S1  14-3-3 and actin are present at similar levels. (A) Coomassie-stained gel showing recombinant GST-cleaved Gl-14-3-3 that was used for quantitative Western blotting. (B) Sypro ruby-stained gel showing TS-actin purified from *Giardia* that was used as a standard for quantitative Western blotting. (C) Representative Western blot measuring the amount of endogenous Gl-14-3-3 in 10 µg of *Giardia* extract (102.5 ± 7.4 ng). (D) Representative Western blot measuring the amount of endogenous Gl-actin in 10 µg of *Giardia* extract (70.7 ± 16.4 ng). Download FIG S1, PDF file, 1.8 MB. Copyright © 2017 Krtková et al. 2017 Krtková et al. This content is distributed under the terms of the Creative Commons Attribution 4.0 International license.

Since 14-3-3 has a role in regulating cell division in other eukaryotes, we examined the localization of an endogenously hemagglutinin (HA) C-terminally-tagged version of Gl-14-3-3 (Gl-14-3-3–HA) (19). (See Fig. 1B for a diagram of *Giardia* cellular landmarks.) In interphase cells, Gl-14-3-3 was distributed throughout the cell with some enrichment at the cortex, perinuclear region, and in association with the intracytoplasmic axonemes of all flagella, but was most apparently associated with the anterior flagella (Fig. 1C; see Fig. S2 in the supplemental material). In mitotic cells, Gl-14-3-3 disassociated with the intracytoplasmic axonemes and enrichment of 14-3-3 were observed around the spindle which may reflect association with the perinuclear membrane/nuclear envelope (Fig. S2). Notably, we previously demonstrated a central role for actin in positioning the flagella and nuclei (21). Gl-14-3-3 was also associated with the ingressing furrow, which does not utilize a contractile ring (Fig. 1C). We recently reported that Gl-actin levels are reduced just ahead of the advancing furrow cortex, and Gl-actin is required for abscission but not furrow progression (24). Enrichment of Gl-14-3-3 just ahead of the furrow cortex may indicate a negative actin regulatory function for Gl-14-3-3 and/or a role in regulating membrane trafficking (Fig. 1C). Consistent with 14-3-3 having a role in regulating membrane trafficking (6), Gl-14-3-3–HA is associated with the nuclear envelope/endoplasmic reticulum (ER) and the bare area of the ventral disc (Fig. 1D). This void in the disc lacks microtubules and serves as a conduit for vesicle trafficking, whereas the rest of the disc is composed of a sheet of microtubules and associated proteins that would physically prevent vesicle transport (25). Although our images indicate partial colocalization between Gl-14-3-3 and Gl-actin, using an antibody that recognizes both monomeric and filamentous Gl-actin (F-actin), we did not observe Gl-14-3-3–HA to colocalize with F-actin structures. This result is consistent with our finding that Gl-14-3-3 complexes with monomeric actin.

10.1128/mSphere.00248-17.2 FIG S2  14-3-3 is associated with axonemes and the perinuclear space. (A) 14-3-3 (magenta) and the ER marker PDI2 (green) localized in an interphase trophozoite (maximal projection of 3 optical sections from the center of the cell). 14-3-3 is associated with the internal axonemes of the anterior flagella (af) and posteriolateral flagella (plf). (B) 14-3-3 (magenta) and tubulin (green) localized in a mitotic trophozoite (maximal projection of 3 optical sections from the center of the cell). 14-3-3 remains associated with the perinuclear region, and localization overlaps with the spindle, which is intimately associated with the nuclear envelope in *Giardia*. Note that 14-3-3 is no longer observed in association with flagellar axonemes. Scale bar, 5 µm. Download FIG S2, PDF file, 0.7 MB. Copyright © 2017 Krtková et al. 2017 Krtková et al. This content is distributed under the terms of the Creative Commons Attribution 4.0 International license.

**FIG 2:**
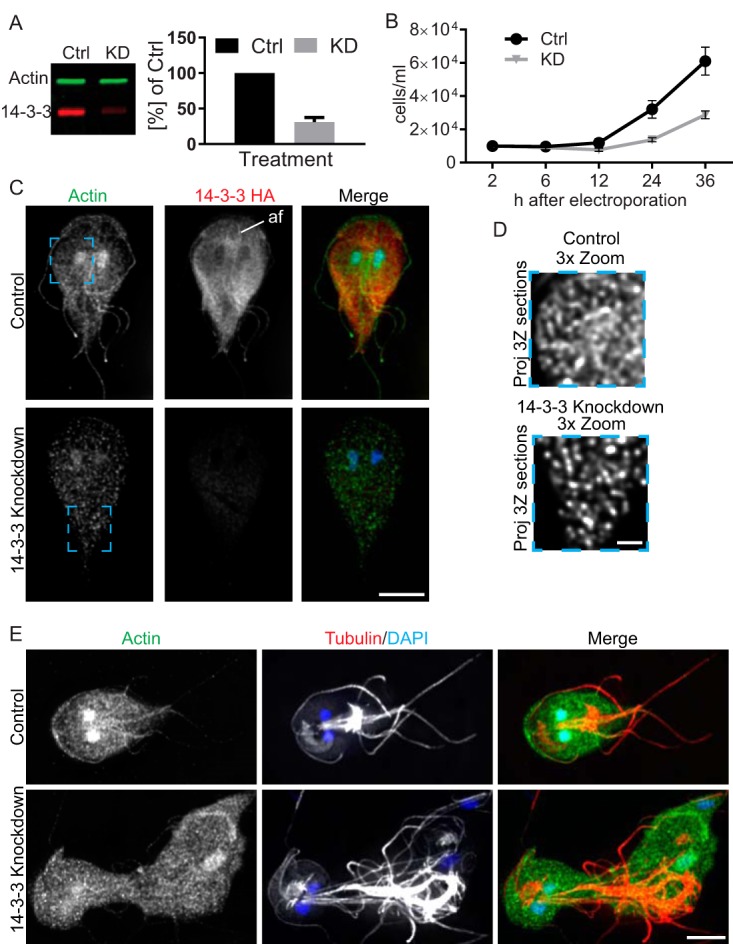
14-3-3 is required for *Giardia* actin cytoskeletal organization and growth. (A) Multiplexed immunoblot showing typical Gl-14-3-3–HA reduction 24 h after morpholino treatment and quantification of three independent experiments. (B) Growth curves of control (Ctrl) and Gl-14-3-3-depleted cell cultures indicate that Gl-14-3-3 is critical for *Giardia* culture growth (Error bars represent standard deviation [SD].) (C) Immunofluorescence staining of control and Gl-14-3-3-depleted cells scaled equally. Note enrichment of Gl-14-3-3–HA along the intracytoplasmic axonemes of the anterior flagella (af) and that depletion of Gl-14-3-3 altered actin organization. Scale bar, 5 µm. (D) Magnified view of the blue box in panel C optimally scaled to show actin filaments in the control and Gl-14-3-3-depleted cells. The puncta in Gl-14-3-3-depleted cells are short filaments; see Movie S1 for an entire image stack. Scale bar, 1 µm. (E) Actin (green) and tubulin (red) staining shows 14-3-3-depleted cells lose cell polarity and have cytokinesis defects. See Fig. S3 for further examples of knockdown phenotypes. Scale bar, 5 µm.

To ascertain whether Gl-14-3-3 has a role in cytoskeletal regulation in *Giardia*, we depleted Gl-14-3-3 with an antisense translation-blocking morpholino. Knockdown (KD) of Gl-14-3-3 protein expression was monitored by immunoblotting to detect the integrated copy of Gl-14-3-3–HA. On average, a 70% reduction in Gl-14-3-3–HA levels was observed 24 h after morpholino treatment, and parasite growth was dramatically reduced in the knockdown population versus the nonspecific morpholino control, indicating a key role in cell proliferation (Fig. 2A and B). Depletion of Gl-14-3-3 disrupted characteristic actin organization and resulted in small bright puncta distributed throughout the cell (Fig. 2C; see Fig. S3 in the supplemental material). Detailed examination revealed that the puncta are short filaments below 1 µm in length (Fig. 2D; see Movie S1 in the supplemental material). Depletion of Gl-14-3-3 also led to polarity and cytokinesis defects, indicating that Gl-14-3-3 has a role in regulating both actin and tubulin cytoskeletal organization (Fig. 2E; Fig. S3). The loss of cell polarity, accumulation of multinucleate cells, reduction in cell growth, and abnormal flagellar positioning associated with Gl-14-3-3 depletion are phenotypes that overlap those observed in Gl-actin-depleted *Giardia* (21). These results likely reflect misregulation of cytoskeletal dynamics which are exquisitely controlled in other eukaryotes. Indeed, drugs which stabilize or depolymerize cytoskeletal arrays often lead to similar defects; therefore, these results are consistent with 14-3-3 having an actin regulatory role in *Giardia*.

10.1128/mSphere.00248-17.3 FIG S3  14-3-3 depletion disrupts the actin cytoskeleton and cellular organization. (A) Actin (green), Gl-14-3-3–HA (red), and DNA (blue) were stained after Gl-14-3-3 depletion; scaling was normalized by exposure time. The top row shows a representative control cell treated with nonspecific morpholino. The bottom rows show that Gl-14-3-3 depletion resulted in aberrant actin organization and cytokinesis defects, such as failure to divide and multinucleate cells. (The wild type has 2 nuclei.) (B) Actin (green), tubulin (red), and DNA (blue) were stained after Gl-14-3-3 depletion; scaling is arbitrary. The top row shows a typical control cell treated with nonspecific morpholino control. The bottom rows show Gl-14-3-3 depletion resulted in cells with aberrant actin organization and cytokinesis defects, such as failure to divide and multinucleate cells (The wild type has 2 nuclei.) Scale bar, 5 µm. Download FIG S3, PDF file, 2.2 MB. Copyright © 2017 Krtková et al. 2017 Krtková et al. This content is distributed under the terms of the Creative Commons Attribution 4.0 International license.

10.1128/mSphere.00248-17.6 MOVIE S1  Image stack showing actin organization in the control and 14-3-3-depleted cells shown in Fig. 2. Download MOVIE S1, AVI file, 0.2 MB. Copyright © 2017 Krtková et al. 2017 Krtková et al. This content is distributed under the terms of the Creative Commons Attribution 4.0 International license.

**FIG 3:**
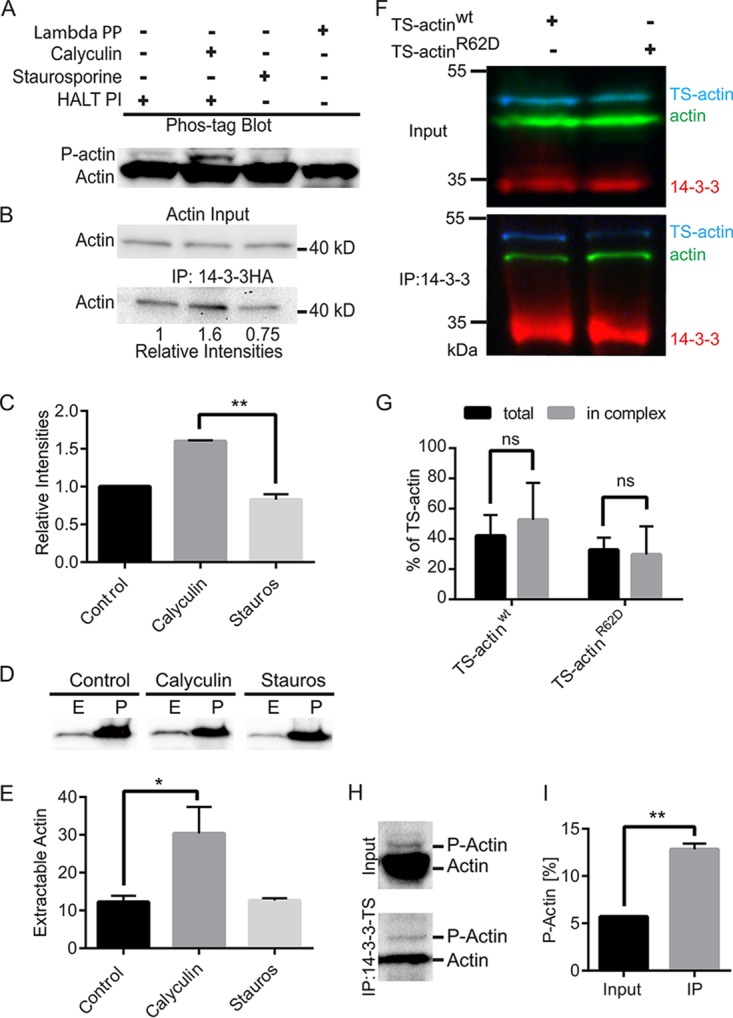
14-3-3–actin complex formation is phospho-dependent. (A) Immunoblot after Phos-tag phosphate-affinity electrophoresis. Cells were pretreated with DMSO or inhibitors and then HALT phosphatase inhibitor (HALT PI) was added at lysis to preserve the phosphorylation state. Calyculin A treatment increased phosphorylated-actin (P-actin) levels, and the kinase inhibitor staurosporine reduced P-actin. Phospho-isoforms were removed after lambda protein phosphatase (PP) treatment. (B) Immunoprecipitation of Gl-14-3-3–HA after calyculin A treatment led to increased actin interaction, while staurosporine treatment reduced the association of actin with Gl-14-3-3–HA. (C) Mean values of three independent experiments. Error bars represent SD. **, *P* < 0.01. (D) Detergent-extractable actin is increased by calyculin A treatment. E, extracted, predominantly G-actin; P, cell pellet/nonextracted, predominantly F-actin. (E) Plots are mean percentages of extractable actin from three independent experiments. Error bars represent SD. *, *P* < 0.05. (F) Pulldown of 14-3-3 in cells expressing wild-type TS-actin or the polymerization-defective R62D mutant. (G) Graph showing binding of wild-type TS-actin compared to the R62D polymerization-defective mutant in three independent experiments. ns, not statistically significant. (H) Compared with input, eluted protein from 14-3-3–TS pulldown shows enrichment of P-actin. (I) Quantification of three independent experiments. **, *P* < 0.01.

Since actin phosphorylation occurs in several eukaryotes (26–32) and 14-3-3 binding usually requires Ser/Thr target phosphorylation, the effect of kinase and phosphatase inhibitors on Gl-actin/Gl-14-3-3 complex stability was studied. The Ser/Thr phosphatase inhibitor calyculin A and the general kinase inhibitor staurosporine are both effective in *Giardia* (33), likely affecting the phosphorylation state of multiple proteins. Using Phos-tag phosphate-affinity electrophoresis (34), we find that a portion of actin is indeed phosphorylated in *Giardia* extracts (Fig. 3A), suggesting phosphorylation could be an important actin regulatory mechanism. After 45 min of treatment with either staurosporine or calyculin A, the level of Gl-actin phosphorylation increased following treatment with the phosphatase inhibitor and decreased as a result of treatment with the kinase inhibitor, respectively (Fig. 3A). Treatment of the cell extracts with lambda protein phosphatase effectively depletes the shifted band, demonstrating the specificity of our actin antibody. Remarkably, our ability to coimmunoprecipitate Gl-actin with Gl-14-3-3–HA correlated with the phosphorylation level of Gl-actin and is consistent with phospho-dependent regulation of the 14-3-3–actin interaction (Fig. 3B and C).

Next we asked whether modulating the phosphorylation level of actin could affect the balance between F- and G-actin. Actin extraction assays were performed after treatment of parasites with staurosporine or calyculin A. Phosphatase inhibition with calyculin A increased extractable Gl-actin from 12.2% in dimethyl sulfoxide (DMSO)-treated control cells to 30.4% (*n =* 3; *P* < 0.05). No reduction in extractable actin was observed after treatment with the kinase inhibitor staurosporine (Fig. 3D and E), possibly due to the limited sensitivity of this assay coupled with our observation that only a small pool of actin is free to begin with. These results do show that actin phosphorylation is correlated with a shift in the balance toward soluble, presumably, G-actin and association with Gl-14-3-3.

The increased association of Gl-14-3-3 with actin that results from calyculin A treatment could indirectly result from increased monomeric actin levels. Therefore, we sought to assess whether increased monomeric actin in the absence of increased phosphorylation could promote complex formation with Gl-14-3-3. Mutation of Arg 62, a key residue also conserved in Gl-actin, to Asp (R62D) has been shown to result in polymerization-defective β-actin (35). To monitor the mutant Gl-actin isoform, TS-actin was mutated and transformed into a Gl-14-3-3–HA-expressing parasite line. In Gl-14-3-3–HA immunoprecipitation, both TS-actin^R62D^ and control TS-actin coprecipitated in similar ratios compared with endogenous Gl-actin (Fig. 3F and G). This result suggests that the association between Gl-actin and Gl-14-3-3 promoted by calyculin depends on increased Gl-actin phosphorylation rather than an increased amount of monomeric Gl-actin. To confirm that Gl-14-3-3 complexes with phosphorylated actin, pulldowns of 14-3-3–TS were performed and the phosphorylation state of the associated actin was assessed with Phos-tag gels and Western blotting. Indeed, compared with input, the phosphorylated forms of actin were enriched in 14-3-3–TS pulldown (Fig. 3H); however, most of the coimmunoprecipitated actin was not phosphorylated. The presence of both phosphorylated and unphosphorylated actin in association with Gl-14-3-3 suggests multiple modes of 14-3-3 association, which could include both direct binding and recruitment through actin-binding proteins.

**FIG 4:**
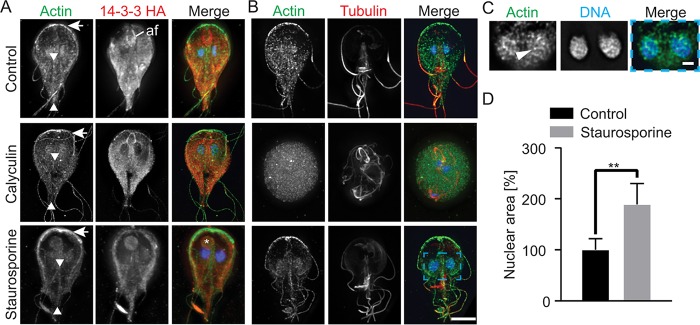
Filamentous actin structures are depleted by calyculin A treatment and enhanced by staurosporine treatment. (A) Projected images of actin (green), Gl-14-3-3–HA (red), and DNA (blue) in the presence of calyculin A and staurosporine. Arrows mark the anterior of the cell where actin intensity is reduced by calyculin A treatment (increased phosphorylation) but enhanced by staurosporine treatment (reduced phosphorylation). Arrowheads mark the intracytoplasmic caudal flagella axonemes, which are typically associated with actin. Note that calyculin A treatment resulted in loss of actin association with this structure, while staurosporine treatment increased actin association. Calyculin A treatment enriched Gl-14-3-3–HA along the intracytoplasmic axoneme of the anterior flagella (af). An asterisk marks the aberrant structure found in 30% of staurosporine-treated cells. Note this structure is associated with the bare region of the disc, a conduit of cellular trafficking. (B) Projected images of actin (green), tubulin (red), and DNA (blue) in the presence of calyculin A and staurosporine. Note that 27% of calyculin A-treated cells lost cytoskeletal organization and adopted a rounded cell shape. Scale bar, 5 µm. Nuclear area increased after staurosporine treatment. (C) A single optical section enlarged from the blue box in panel B showing actin filaments associated with the nuclei. An arrowhead marks a prominent filament. (D) Nuclear area quantified after treatment with staurosporine (control [DMSO], *n =* 22; staurosporine, *n =* 64). Values are means ± SD. **, *P* < 0.01. Scale bar, 1 µm.

Since modulation of Gl-actin phosphorylation changed the balance between F- and G-actin, we anticipated that this would be apparent as changes in cellular actin organization. To verify this hypothesis, cells were treated with calyculin A or staurosporine for 30 min and then stained for Gl-actin and Gl-14-3-3–HA or tubulin. Treatment with the phosphatase inhibitor calyculin A resulted in an apparent decrease in the robustness of actin structures and enrichment of Gl-14-3-3 along the intracytoplasmic axonemes of the anterior flagella (Fig. 4A). More severely impacted cells (27% [83/300]) lost cytoskeletal organization and became spherical (Fig. 4B). Conversely, treatment with staurosporine led to increased cortical actin and brightly labeled F-actin structures, the most apparent of which are at the anterior of the cell (Fig. 4A). Prominent actin filaments associated with the nuclei of staurosporine-treated cells were also apparent (Fig. 4B and C). In 30% (90/300) of staurosporine-treated cells, we observed an aberrant structure containing actin and 14-3-3 in proximity to the bare area of the ventral disc, suggesting that membrane trafficking is impaired (Fig. 4A, asterisk). Nuclear size was also increased. Staurosporine treatment increase nuclear area by 89% compared to DMSO-treated control cells, and prominent nuclear actin filaments were observed to span the entire width of the nuclei (*P* < 0.001; *n =* 43 for control, and *n =* 32 for staurosporine treated). This phenotype suggests that actin may regulate nuclear size in *Giardia*; alternatively, staurosporine treatment caused the nuclei to swell and actin filaments grew to occupy the available space. Overall, phosphorylation of the cytoskeleton appears to be correlated with Gl-14-3-3 association and cytoskeletal disassembly, while inhibition of phosphorylation with staurosporine stabilized cytoskeletal structures.

**FIG 5:**
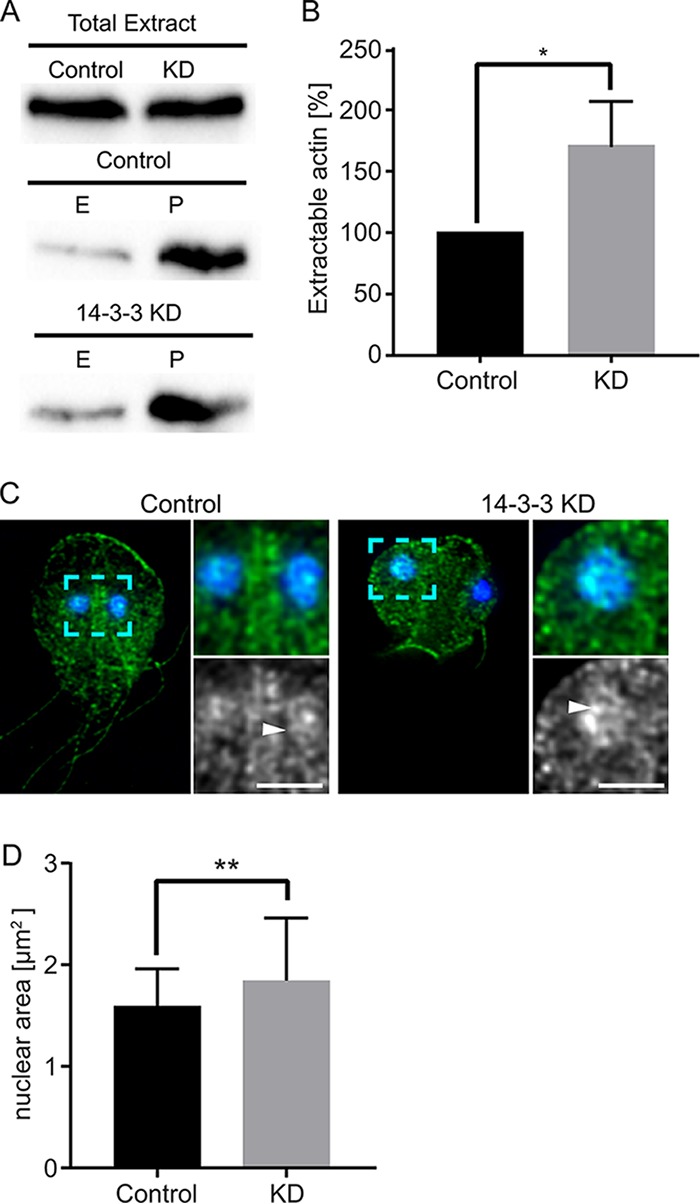
14-3-3 knockdown alters actin extractability and increases nuclear size**.** (A) Detergent-extractable actin is increased by 14-3-3 knockdown (KD). E, extracted; P, cell pellet/nonextracted. (B) The plot is normalized to extracted actin from the matching control; results are from three independent experiments. Error bars represent SD. *, *P* < 0.05. (C) Actin (green) and DAPI (blue) staining from control and 14-3-3 KD. Nuclear size is variable in 14-3-3 KD cells. A magnified view (dashed cyan box) shows that although filaments are smaller in the cytoplasm, the large nuclei have robust filaments that span the width of the nuclei. (The white arrowhead points to the nuclear actin filament, which is also apparent in Fig. 2E.) Scale bar, 2 µm for magnified views. (D) Quantification of nuclear area indicates a 16.4% average increase in 14-3-3 KD cells compared to controls (control, *n =* 60; KD, *n =* 96). **, *P* < 0.01.

Considering the differences in actin extractability and changes in nuclear size associated with staurosporine and calyculin A treatment, we questioned whether Gl-14-3-3 depletion might also alter actin extractability and nuclear size. In Fig. 2D, we showed that depletion of Gl-14-3-3 led to ectopic actin distribution: the small puncta appear to be short filaments that could result from changes in actin regulation such as spontaneous actin nucleation or reduced filament stability. The amount of actin extracted from the Gl-14-3-3 knockdown cells increased in comparison to the control in each of the three replicates (Fig. 5A and B). This result confirms that Gl-14-3-3 depletion alters the state of actin. However, due to the presence of short actin filaments, it is not clear if the increased extractability is due to increased globular actin or an indication that the short filaments are simply more extractable than the larger filaments found in control cells. We examined nuclear area in 14-3-3 knockdown cells and found that like staurosporine treatment, nuclear area was increased, although to a lesser extent (Fig. 5C and D). Intriguingly, while actin filaments in the cytoplasm appear smaller in 14-3-3 knockdown cells, actin filaments associated with nuclei are more robust and are reminiscent of those observed in staurosporine-treated cells. These results are consistent with 14-3-3 regulating both the actin cytoskeleton and nuclear size.

**FIG 6:**
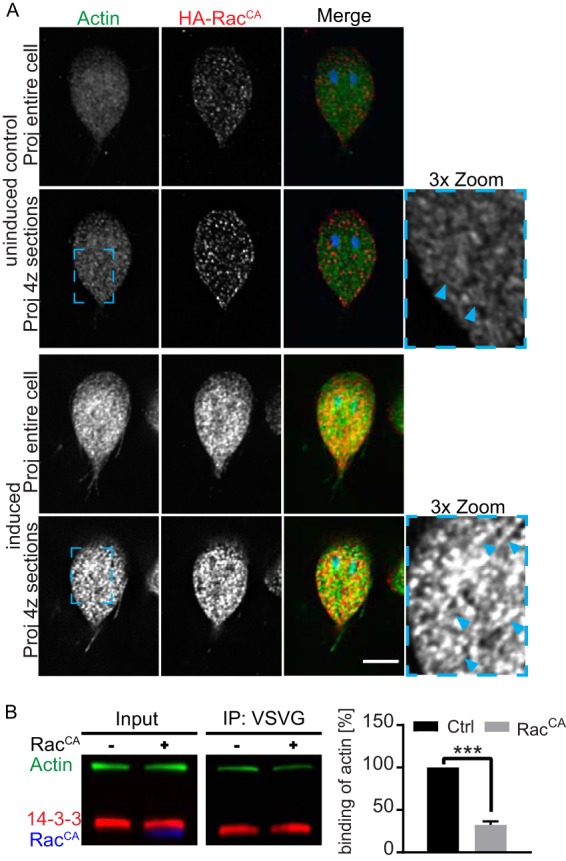
Rac signaling modulates 14-3-3–actin complex formation. (A) Actin filaments (green) are more prominent in *tet*-induced HA-Rac^CA^-expressing cells. Note that the *tet* promoter is leaky and some expression is detected in uninduced control cells (images scaled equally). (B) Immunoprecipitation of actin with 14-3-3–VSVG from uninduced (−) and induced (+) HA-Rac^CA^ cell lines and quantification of actin binding from three independent experiments. ***, *P* < 0.001. Scale bar, 5 µm.

Next we questioned whether, Rho GTPase signaling could modulate 14-3-3–actin complex formation. Expression of an inducible N-terminally HA-tagged constitutively active Q74L Gl-Rac (HA-Rac^CA^; equivalent to Q61L Rac1) was previously observed to increase overall actin fluorescence, which we suggested was due to increased filament formation (21). Indeed, detailed analysis revealed the formation of prominent Gl-actin filaments at the cell cortex (Fig. 6A). Induction of the HA-Rac^CA^ mutant protein led to a decrease in the amount of actin that coimmunoprecipitated with vesicular stomatitis virus G glycoprotein (VSVG) tagged 14-3-3, suggesting that Gl-Rac inhibits 14-3-3–actin complex formation (Fig. 6B). This result provides the first link between Rho GTPase signaling and actin in *Giardia*, since all of the proteins that normally link Rho GTPases and the actin cytoskeleton are missing.

**FIG 7:**
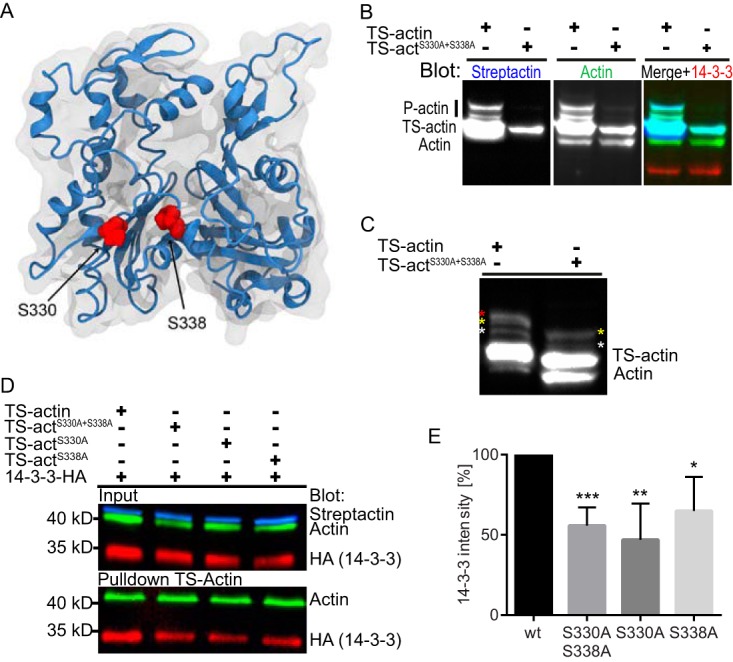
S330 and S338 of Gl-actin contribute to 14-3-3 complex formation. (A) Model of Gl-actin showing the positions of S330 and S338 in an actin monomer. (B) Multiplexed immunoblot of total *Giardia* extracts after Phos-tag phosphate-affinity electrophoresis comparing phosphorylation of TS-actin and TS-actin^S330A S338A^; anti-Gl-actin (green), StrepTactin-HRP (blue), and anti-HA (red). Note equal loading as indicated by 14-3-3 levels. (C) Samples from panel B were overloaded and probed with anti-Gl-actin antibody. Colored asterisks mark specific P-actin bands: note that only two bands are visible in the TS-actin^S330A S338A^ double mutant. (D) Affinity pulldown of TS-actin variants blotted for Gl-actin and 14-3-3–HA. (E) Quantification of three independent affinity pulldown experiments shows S330 and S338 contribute to 14-3-3 association. *, *P* < 0.05; **, *P* < 0.01; ***, *P* < 0.001.

After demonstrating that 14-3-3–actin complex levels could be modulated by Rho GTPase signaling and drug treatments, we sought to determine if there are binding motifs in Gl-actin that could support direct interaction with Gl-14-3-3. The 14-3-3-Pred prediction algorithm tool identified S330 (RVRIpSSP) and S338 (RKYpSAW) as the highest-scoring predicted interaction sites (36). In agreement with this finding, the same sites were previously reported using a custom algorithm to identify binding sites in putative *Giardia* 14-3-3 interactors (22). These sites are similar to the canonical mode 1 site RXXp(S/T)XP, where p(S/T) are phosphorylated serine or threonine residues; while not a perfect match, many 14-3-3-interacting proteins have been found that lack canonical mode 1-3 binding motifs (3, 36, 37). To assess the potential involvement of these sites, shown as surface accessible (Fig. 7A), TS-actin was mutated to generate an S330A S338A double mutant. The wild-type and mutant TS-actin constructs were introduced into the endogenously tagged Gl-14-3-3–HA parasite line. Phos-tag gel analysis of the S330A S338A double mutant confirms that at least one of these two sites is phosphorylated (Fig. 7B and C). The double mutant as well as single-point mutants had reduced capacity to coprecipitate 14-3-3–HA (Fig. 7D). The mean reductions in 14-3-3 binding were similar for the S330A and S338A single mutants and the S330A S338A double mutant. The double mutant, however, showed more consistent reduction, as noted by error bar size in Fig. 7E. Incomplete disruption of the Gl-14-3-3 interaction could indicate that part of the 14-3-3 recruitment is mediated through association with ABPs. This further raises the possibility that the point mutations that reduced complex formation with 14-3-3 could cause structural changes that reduce interaction with ABPs which recruit 14-3-3. Alternatively, the incomplete disruption of 14-3-3–actin complex formation could indicate the presence of additional interaction sites. Thus, we also tested the possible involvement of T162 (VTHpTVP), a conserved residue identified by Scansite 3 as the highest-scoring 14-3-3 interaction site (38). However, mutation of T162 to alanine did not disrupt 14-3-3–actin interaction, consistent with structure homology modeling that suggested this residue was not surface accessible (see Fig. S4 in the supplemental material). These results are in line with S330 and S338 having a role in promoting 14-3-3–actin complex formation. However, our inability to completely disrupt actin complex formation indicates additional means for 14-3-3’s association with actin.

10.1128/mSphere.00248-17.4 FIG S4  T162 does not contribute to 14-3-3 binding. (A) Model of an actin filament with T162 and S338 indicated. (B) Front and back views of modeled actin monomers indicating the positions of T162 and S338. Note that T162 is completely inaccessible in filaments, since it is between the two filament strands and almost entirely buried in the G-actin monomer (C) Pulldowns from cells expressing wild-type and mutated (T162A, S338A) TS-actin. Note that TwinStrep-actin was detected in the input with StrepTactin-HRP: thus, endogenous actin does not appear in this experiment. Download FIG S4, PDF file, 0.3 MB. Copyright © 2017 Krtková et al. 2017 Krtková et al. This content is distributed under the terms of the Creative Commons Attribution 4.0 International license.

**FIG 8:**
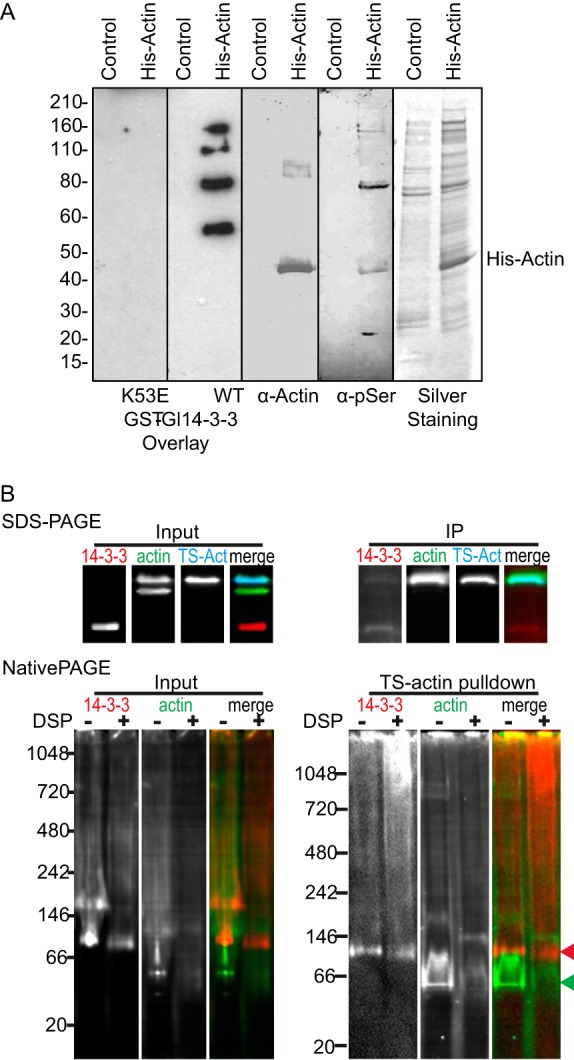
14-3-3–actin complex formation requires intermediate proteins. An immunoblot of affinity-purified 6×His-tagged Gl-actin (His-actin) or mock purification from wild-type trophozoites was assessed by overlay with recombinant GST–Gl-14-3-3 or the 14-3-3 binding-defective mutant GST-K53E. Interaction of GST–Gl-14-3-3 with actin and copurified proteins was revealed by incubation with anti-GST–HRP. The same membrane was stripped and probed with mouse anti-Gl-actin and again with mouse MAb anti-pSer. Silver-stained protein purifications are shown in the last inset. Molecular mass markers (kilodaltons) are on the left. The position of His-actin is indicated on the right. (B) Affinity-purified TS-actin was run on SDS-PAGE and native PAGE. The native gel analysis includes DSP-cross-linked samples to preserve native complexes. Note that DSP treatment reduces the amount of ~100-kDa dimeric 14-3-3 (red arrowhead) and increases the high-molecular-mass smear. The position of putative monomeric TS-actin (45.2-kDa expected size) is marked with a green arrowhead.

To test whether Gl-14-3-3 can directly bind Gl-actin, overlay experiments were performed using a 6×His-Gl-actin expressed in and purified from *Giardia* extracts to ensure native phosphorylation. The overlay was performed with either the recombinant wild-type glutathione *S*-transferase (GST)-fused Gl-14-3-3 or with the mutant GST-K53E, previously shown to be binding defective (39, 40). Binding of GST–Gl-14-3-3 near the molecular mass of 6×His-Gl-actin was not observed (Fig. 8A). Instead, GST–Gl-14-3-3 bound to four bands on the blot corresponding to proteins copurified with 6×His-Gl-actin. The most prominent labeling is associated with a major copurified protein at 80 kDa also seen in silver staining. Immunoblotting with an anti-pSer antibody confirmed that a fraction of actin was phosphorylated, as well as some copurified proteins, including the 80-kDa protein band (Fig. 8A). This result confirms that some of the 14-3-3–actin interaction is due to recruitment through additional binding partners that can mediate the formation of a complex containing both Gl-14-3-3 and Gl-actin. The inability to bind directly to actin could reflect a requirement for actin to be natively folded or that the interaction site is low affinity and additional proteins are needed to stabilize binding (41).

To assess whether *Giardia* contains a 14-3-3 complex appropriately sized for direct interaction, we turned to native gel electrophoresis. If a 14-3-3–actin complex formed without any additional proteins, it would contain a dimer of 14-3-3 (42) and a single TS-actin monomer. Previous native gel analysis indicated that 14-3-3 dimers run at ~80 kDa (42) and TS-actin is 45.2 kDa; taking into account the size of the 3×HA tag (3.8 kDa) the anticipated complex size for direct binding is around 130 kDa. TS-actin and associated proteins were purified, immediately run on native gels, and transferred to membrane for Western blotting. A prominent band of Gl-14-3-3 was detected running between the 66- and 140-kDa native gel markers (Fig. 8B). However, a similarly sized band is also apparent in the input, suggesting this band represents free 14-3-3 dimer and that the complexes were unstable. In an effort to stabilize actin complexes, we used the membrane-permeable cross-linker DSP to cross-link samples before lysis. Cross-linking did change the distribution of actin, and a prominent actin band running just below 146 kDa was enriched in the pulldown (Fig. 8B); however, a corresponding enrichment of 14-3-3 was not apparent. Instead, the larger-molecular-mass smear of Gl-14-3-3 became more prominent, consistent with multiple actin interactors having the capacity to bind actin. Future work will be required to identify these 14-3-3–actin-interacting proteins and determine their specific roles in regulating the Gl-actin cytoskeleton. Although the relationship between Gl-14-3-3 and actin appears to be more complicated than we initially imagined, our results indicate that Gl-14-3-3 plays a decisive role in actin regulation.

## DISCUSSION

Proteomic studies support complex formation between 14-3-3 and actin without the involvement of cofilin (10–12, 22). A recent study of 14-3-3σ function in the basal-like progression of breast cancer cells found that 14-3-3σ forms a complex containing actin and two intermediate filament proteins (13). The 14-3-3σ complex sequesters soluble actin in a bioavailable form that is then used for directional assembly of the cytoskeleton during cell migration. Similarly, *Giardia* appears to use 14-3-3 to restrict actin assembly to specific subcellular regions, as evidenced by the 14-3-3 knockdown experiments where actin filaments were dispersed throughout the cell and typical actin organization was lost.

We estimated the intracellular concentration of actin to be around 4.7 µM. This relatively low concentration is nevertheless above the critical concentration for Gl-actin (21), indicating the need for actin sequestration. The emerging view of actin network homeostasis is that sequestering proteins play an important role in partitioning actin to competing F-actin networks (43, 44). In other eukaryotes, the concentration of sequestering proteins exceeds the actin monomer pool (45), but *Giardia* lacks all known monomer sequestering proteins. Our results indicate that Gl-14-3-3 is associated with monomeric actin in complexes with other interaction partners (Fig. 7) and that complex formation has a reverse correlation with actin filament formation (Fig. 3, 4, and 6). Therefore, it seems likely that 14-3-3 functions in the partitioning of actin complexes for subfunctionalization in addition to having a role in maintenance of G/F-actin homeostasis.

Complex formation between Gl-14-3-3 and Gl-actin appears to be both dependent and independent of phosphorylation. The putative 14-3-3 phospho-binding motif centered on S338 is conserved in mammalian actin and has previously been implicated as an AKT phosphorylation site (46). The 14-3-3 binding site prediction tool 14-3-3-Pred identified S338 as the highest-scoring site for both Gl-actin and human β-actin. We have shown that mutation of this site as well as S330 reduces 14-3-3 recruitment, and at least one of these predicted interaction sites is phosphorylated. Our ability to perform biochemical assays with *Giardia*’s highly divergent actin remains limited. Overlay assays and native gels did not provide support for direct interaction between Gl-14-3-3 and Gl-actin. However, it remains possible that some of the observed complexes contain Gl-14-3-3 directly bound to Gl-actin with additional interactors that work to stabilize the complex (41). Indeed, the 14-3-3σ complex contains two intermediate filament proteins, yet *in vitro* actin polymerization assays containing only actin and 14-3-3σ demonstrated that 14-3-3 could directly regulate actin dynamics (13). The need for additional proteins to stabilize the 14-3-3 interaction with S330 and S338 would reconcile our data; however, a major caveat is that these point mutants could have disrupted Gl-actin folding and therefore disrupted the binding of proteins that recruit Gl-14-3-3.

While we have yet to pursue proteins that associate with both Gl-14-3-3 and Gl-actin, independent proteomic studies aimed at identifying 14-3-3 and actin interactors point toward proteins of interest (19, 22). Besides Gl-actin and Gl-14-3-3, there are 14 proteins in common between the two studies (see Table S1 in the supplemental material). Eight of these proteins belong to the highly conserved chaperonin containing TCP-1 (CCT; molecular mass, 56.3 to 64.7 kDa), which is known to have a role in actin folding (47). The TCP-1 epsilon subunit has been implicated in the regulation of actin dynamics and this subunit has two, albeit untested, canonical mode 1 14-3-3 recognition motifs (22, 48). Intriguingly, the epsilon subunit was the most abundant component of the TCP-1 complex found in our actin interactome. The list also includes TIP49 (GL50803_9825 [51.4 kDa]), which we have validated as a robust actin interactor through reciprocal pulldown (19). Other proteins include an SMC domain protein (GL50803_6886 [102.8 kDa]), two dynein heavy-chain proteins (GL50803_111950 [570.3 kDa] and GL50803_42285 [834.7 kDa]), and two proteins without any conserved domains (GL50803_15251 [32.5 kDa] and GL50803_15120 [26.6 kDa]). All of these proteins have at least one canonical mode 1 14-3-3 recognition motif, but whether they are involved in recruiting 14-3-3 to actin complexes remains to be determined.

10.1128/mSphere.00248-17.5 TABLE S1  Intersection between Gl-actin and Gl-14-3-3 interactome studies. Download TABLE S1, DOCX file, 0.01 MB. Copyright © 2017 Krtková et al. 2017 Krtková et al. This content is distributed under the terms of the Creative Commons Attribution 4.0 International license.

Overall, our results support a role for Gl-14-3-3 associating with monomeric Gl-actin complexes that are downstream of Gl-Rac, where phosphorylated actin and phosphorylated actin interactors are held inactive. This work illustrates the conserved role of 14-3-3 as an actin regulator, albeit through an alternative set of actin-binding proteins that remain to be identified and validated.

## MATERIALS AND METHODS

### Strain and culture conditions.

*Giardia intestinalis* strain WBC6 was cultured as described in reference 49.

### Culture and microscopy.

Fixations were performed as described in reference 21. Anti-HA HA7 (Sigma), anti-tubulin 11-B6-1 (Sigma), anti-Gl-actin 28PB+1 (21), and anti-PDI2 (50) antibodies were used at 1:125, and secondary antibodies were used at 1:200 (Molecular Probes). Isotype-specific anti-mouse secondary antibodies (Molecular Probes) were used for colocalization of tubulin (IgG2b) and HA (IgG1). PDI2 and HA mouse antibodies were colocalized using Zenon labeling (Molecular Probes). Images were acquired on a DeltaVision Elite microscope using a 100 × 1.4 NA objective and a Coolsnap HQ2 or PCO EDGE sCMOS camera. Deconvolution was performed with SoftWorx (GE, Issaquah, WA). Average and maximal projections were made with ImageJ (51), and figures were assembled using Adobe Creative Suite (Mountain, CA). Nuclear area measurements were made from thresholded maximal projections of DAPI (4′,6-diamidino-2-phenylindole) staining using ImageJ.

### Morpholino and drug studies.

Morpholino treatments with anti-14-3-3 CGCGTAAATGCCTCGGCCATAGGTT and control CCTCTTACCTCAGTTACAATTTATA were performed as described in reference 52. Calyculin A and staurosporine (LC Laboratories, Woburn, MA) were diluted in DMSO and used at 1 μM and 200 nM final concentrations, respectively. Cells were treated for 45 min at 37°C.

### Constructs and mutagenesis.

Endogenous tagging of Gl-14-3-3 with 3×HA and construction of N-terminally TS-tagged Gl-actin are described in reference 19. Actin T162A, S330A, and S338A site mutations were introduced into TwinStrep-tagged actin using the QuikChange Lightning multisite-directed mutagenesis kit (Agilent Technologies, Wilmington, DE) using primers S330 (CTGTCCTCGGGACTAGCTATGCGCACACGCTTC), T162 (GACGGGGTGACGCATGCTGTTCCTGTGTAC), and S338 (CGAGGACAGAAAGTACGCTGCCTGGGTTGGTG). Construction of Q74L HA–Gl-Rac (HA-Rac^CA^) under the *tet* promoter is described in reference 21. To construct 14-3-3–VSVG, 14-3-3 (GL50803_6430) was cut from the pKS-6430-3HA_Neo vector (19) using BamHI and AflII and cloned into pKS-VSVG_Neo. pKS-VSVG_Neo was constructed as follows. VSVG tag flanked with 5′ AflII and EcoRI 3′ restriction sites was PCR amplified using VSVG F′ (5′-TGGCTTAAGTATACTGATATTGAAATGAATCGCTTAGGTAAAGGGTCCTACACCGACATCGAGATGAACCGCTTG-3′) and VSVG STOP R′ (5′-TTTGAATTCTCATTTTCCAAGTCTGTTCATTTCTATGTCTGTATAAGAGCCCTTGCCCAAGCGGTTCATCTCGATGTC-3′) overlapping template oligonucleotides and cloned into pKS-3HA.neo to replace the 3×HA tag. A multiple cloning site was introduced into the vector by ligation with annealed linkers MCSlinkerF (5′-GATCCCCCGGGCTGCAGGAATTCGATATCAAGCTTATCGATACCGTCGACCTCGAGC-3′) and MCSlinkerR (5′-TTAAGCTCGAGGTCGACGGTATCGATAAGCTTGATATCGAATTCCTGCAGCCCGGGG-3′). To ensure integration into the genome and endogenous levels of expression, the 14-3-3–VSVG NEO vector was linearized using BsRGI, ethanol (EtOH) precipitated, and transformed into a *Giardia* cell line already containing Q74L HA–Gl-Rac under the *tet* promoter. To construct 14-3-3–TS, 14-3-3 was amplified from genomic DNA using the forward primer OL368 (5′-tatagaatactcaagcttggcgcgccGGAAAATGTGTGATCACCCC-3′) and reverse primer OL369 5′-GCTCCAAGCGCTCCCaccggtCTTCTCCTCGGCATTATCGT-3′), and the product was inserted into p7031 TwinStrepPac digested with AgeI and AscI using Gibson assembly. The 6×His-Gl-actin vector for expression in *Giardia* was generated by PCR amplifying the 6×His tag and coding region from 6×His-actin Bestbac (21) using Primers 408176×His-F NheI (ATGGCTAGCCATCACCATCACCATCACGA) and 408176×His-R ClaI (TGTATCGATAACAATCCCGG). The PCR-amplified insert was ligated into the TwinStrep-actin vector (19) after preparing the vector and PCR product with ClaI and NheI.

### Quantification of cellular Gl-actin concentration.

Trophozoites were counted with a MoxiZ Coulter counter (Orflo), washed 2× with HEPES-buffered saline plus HALT protease inhibitor and phenylmethylsulfonyl fluoride (PMSF), and then boiled in 2% SDS–62.5 mM Tris-HCl (pH 6.8). Protein concentrations in lysates were then quantified by DC assay (BioRad). TwinStrep-tagged actin was purified from *Giardia* trophozoites. Trophozoites were lysed by sonication in buffer consisting of 50 mM Tris-HCl (pH 7.5), 150 mM NaCl, 7.5% glycerol, 0.25 mM CaCl_2_, 0.25 mM ATP, and 0.1% Triton with PMSF and HALT protease inhibitors. Lysates were rotated with StrepTactin resin (IBA) for 2 h at 4°C. Resin was washed a total of four times with modified G buffer (10 mM Tris [pH 8.0], 0.2 mM CaCl_2_, 0.2 mM ATP). Washes 1, 3, and 4 consisted of G buffer plus 500 mM NaCl and 0.1 mM dithiothreitol (DTT). Wash 2 consisted of G buffer plus 500 mM NaCl, 0.1 mM DTT, and 0.1% Tween 20. Protein was eluted with 2.5 mM biotin in G buffer. Western blotting was performed with polyclonal Gl-actin rabbit antibody (21), as detailed below.

### Quantification of cellular Gl-14-3-3 concentration.

Trophozoites were lysed in phosphate-buffered saline (PBS)–1% Triton X-100 (supplemented with protease and phosphatase inhibitors) for 1 h on ice and pelleted for 15 min at 13,000 rpm at 4°C, and supernatant was collected and quantified by Bradford assay. Recombinant GST–Gl-14-3-3–poly(G)_20_ was prepared and purified as previously described (42). Western blotting was performed with anti N-terminal Gl-14-3-3 rabbit antiserum (40) at 1:10,000.

### Detergent-extractable actin.

The detergent-extractable actin assay compares the fraction of Triton X-100-extractable actin versus nonextractable actin within the cell. While some filaments may be extracted by detergent treatment, the detergent-extractable fraction is largely composed of monomeric actin, while actin filaments associated with larger scaffolds remain within the cell (53, 54). Confluent 8-ml cultures were treated for 45 min with drugs, chilled to detach cells, and then pelleted at 700 × *g*. The pellet was resuspended in 800 μl of HBS plus protease inhibitors and moved to a microcentrifuge tube. The cells were pelleted again and then resuspended in 100 μl of actin-stabilizing lysis buffer consisting of 50 mM PIPES [piperazine-N,N′-bis(2-ethanesulfonic acid); pH 6.9], 50 mM NaCl, 5 mM MgCl_2_, 5 mM EGTA, 5% (vol/vol) glycerol, 0.1% Triton X-100, 0.1% Tween 20, 0.1% 2-mercaptoethanol, 0.2 mM ATP, and HALT protease inhibitor. The samples were incubated on ice for 5 min to perforate the membrane and then pelleted at 700 × *g* for 5 min. The supernatants were reserved, and the pellets were resuspended in 80 μl of 8 M urea for 1 h on ice. Equal amounts of each sample were boiled in sample buffer, loaded for SDS PAGE, and transferred to Immobilon-P membrane (Millipore). Actin was detected with the 28PB+1 antibody (1:3,000) and anti-rabbit horseradish peroxidase (HRP)-conjugated secondary antibodies (1:6,000 [BioRad]), Western Lightning Plus ECL enhanced chemiluminescence reagent, and a BioRad Chemidoc MP digital gel doc (with further details provided below).

### Immunoprecipitation and pulldowns.

Immunoprecipitation began with 1 to 3 confluent 13-ml tubes per epitope-tagged cell line. After detachment by icing, cells were pelleted at 700 × *g* and washed once in HBS. The cells were resuspended in 300 μl of lysis buffer (50 mM Tris [pH 7.5], 150 mM NaCl, 7.5% glycerol, 0.25 mM CaCl_2_, 0.25 mM ATP, 0.05 mM DTT, 0.5 mM PMSF, 0.1% Triton X-100, 2× HALT protease inhibitors [Pierce]) and sonicated. The lysate was cleared by centrifugation at 10,000 × *g* for 10 min at 4°C and added to 30 μl of lysis buffer-equilibrated anti-HA (Sigma), anti-VSVG (Sigma), or StrepTactin (IBA) beads. After 1.5 h of binding, the beads were washed four times with wash buffer (25 mM Tris [pH 7.5], 150 mM NaCl, 0.25 mM CaCl_2_, 0.25 mM ATP, 5% glycerol, 0.05% Tween 20) and then boiled in 50 μl of sample buffer. Immunoblotting was performed as described below.

### Phos-tag gels.

After lysis or immunoprecipitation, samples were loaded onto 10% SDS gel supplemented with 100 μM MnCl_2_ and 20 μM acrylamide-pendant Phos-tag AAL-107 (NARD Institute, Ltd. [from 5 mM stock solution in 3% MeOH in distilled water, prepared according to the manufacturer’s instruction]) to detect mobility shift of phosphorylated proteins. The gels were run at 100 V for approximately 2 h. Immunoblotting was performed as described below.

### Native gels.

TS-actin and associated proteins were purified as described above with slight modifications. After cell lysis, the complexes were bound to StrepTactin resin (IBA, Lifesciences, Germany). Unbound complexes were washed out two times, and the 14-3-3–actin complexes were eluted with wash buffer supplemented with 2 mM biotin (Sigma). The samples were dissolved in native PAGE sample buffer (4×) and immediately loaded onto 4 to 16% native PAGE Novex bis-Tris minigel (Life Technologies, Inc.) according to the manufacturer’s instructions. Immunoblotting was performed as described below.

### Western blotting.

After gel electrophoresis, proteins were transferred to Immobilon-FL using wet transfer at 200 mA for 1 h (2 h for Phos-tag and native gels) and blocked in Tris-buffered saline (TBS) plus 5% nonfat dry milk. Immunoprecipitations with the double transformant 14-3-3–VSVG and *tet*-inducible HA-Rac^CA^ cell line were performed after 24 h of 20 µg/ml tetracycline induction. Primary antibody 28PB+1 (actin, rabbit polyclonal) was used at 1:2,500, Sigma HA7 (HA, IgG1) was used at 1:2,500, IBA StrepTactin-HRP was used at 1:7,000, Sigma P5D4 (VSVG, IgG1) was used at 1:1,500, and Abcam, Inc., HA.C5 (IgG3) was used at 1:1,500 and detected with Molecular Probes fluorescent isotype-specific secondary antibodies at 1:2,500. HRP-conjugated secondary antibodies were detected with Western Lightning Plus ECL reagent. Blots were imaged on a Chemidoc MP (BioRad) digital gel doc. Quantification of exported 16-bit TIFF images was performed using ImageJ (51).

### Overlay assays.

*Giardia* protein extracts were prepared from 1 × 10^9^ WBC6 trophozoites or the 6×His-Gl-actin transgenic line. Parasites were recovered by chilling on ice and then washed three times with cold PBS, and the cell pellet was frozen at −70°C overnight. Cells were resuspended in 1 ml of buffer A (50 mM NaH_2_PO_4_, 300 mM NaCl, 10 mM imidazole, 0.005% Tween 20 [pH 8.0]), supplemented with protease/phosphatase inhibitor cocktail (Cell Signaling, Inc., Danvers, MA), lysed by 7 cycles of sonication at 15% power (Sonoplus; Bandelin), and centrifuged at 24,000 rpm at 4°C for 30 min. Supernatant was collected and incubated with 200 µl of Ni-nitrilotriacetic acid (NTA) magnetic agarose bead suspension (Qiagen, Germany) at 4°C for 1 h with gentle rotation. Beads were extensively washed with wash buffer A containing 20 mM imidazole. Bound proteins were eluted in buffer A supplemented with 250 mM imidazole and then dialyzed and concentrated in buffer (50 mM Tris [pH 8.0], 2 mM CaCl_2_, 2 mM ATP). An aliquot (1:4) of purified proteins was separated on 4 to 12% NuPAGE gel in MOPS (morpholinepropanesulfonic acid)-SDS buffer (Invitrogen), blotted on nitrocellulose membrane, and blocked 1 h in 5% nonfat dry milk–HT buffer (20 mM HEPES-KOH [pH 7.6], 75 mM KCl, 5 mM MgCl_2_, 1 mM DTT, 0.1 mM EDTA, 0.04% Tween 20). The membrane was incubated with 10 µg/ml of either GST–Gl-14-3-3 or the GST-K53E mutant (39, 40) in 2.5% nonfat dry milk–HT buffer overnight at 4°C. Protein-protein interaction was assessed by incubation with anti-GST–HRP (1:1,000 [GE Healthcare]) and revealed by chemiluminescence. The membrane was then stripped and reprobed with anti-Gl-actin (1:5,000), and the Western blot was developed using 3,3′-diaminobenzidine (DAB). Alternatively, the membrane was probed with mouse monoclonal antibody (MAb) anti-pSer (1:200 [Sigma-Aldrich]) in 3% bovine serum albumin (BSA)–TBS plus 0.05% Tween 20 (TTBS) buffer, and the Western blot was developed using DAB. Aliquots (1:20) of purified protein were alternatively visualized by silver staining (GE Healthcare).

### Statistical analysis.

For each experiment with a *P* value, we compared at least three biological replicates using a two-tailed *t* test.

### Structure analysis.

We used the Oda et al. F-actin structure (PDB 2ZWH) (55) and the ADP G-actin structure (PDB 1J6Z) (56) to determine the solvent accessibilities of T162, S330, and S338.
