# Supplementary figures and images for: 14-3-3 Regulates Actin Filament Formation in the Deep-Branching Eukaryote Giardia lamblia
Source: mSphere. 2017 Sep 13;2(5):e00248-17. doi: 10.1128/mSphere.00248-17 (PMC5597967; doi:10.1128/mSphere.00248-17)

A

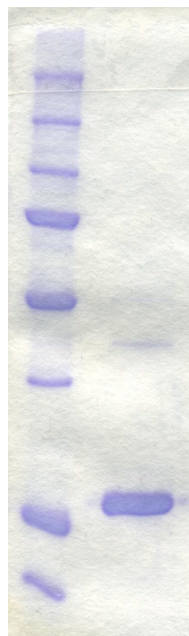

B

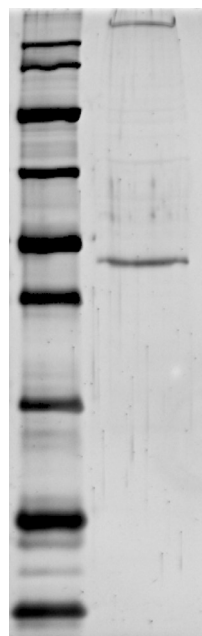

14-3-3

C

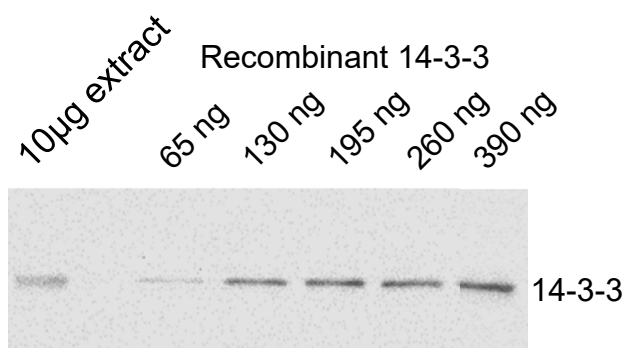

D

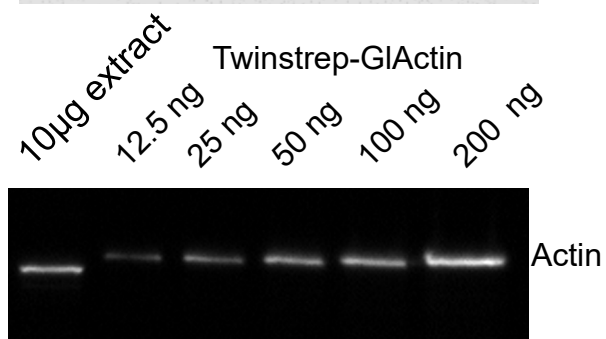

Supplement: FIG S1 [file sph005172359sf1.pdf]

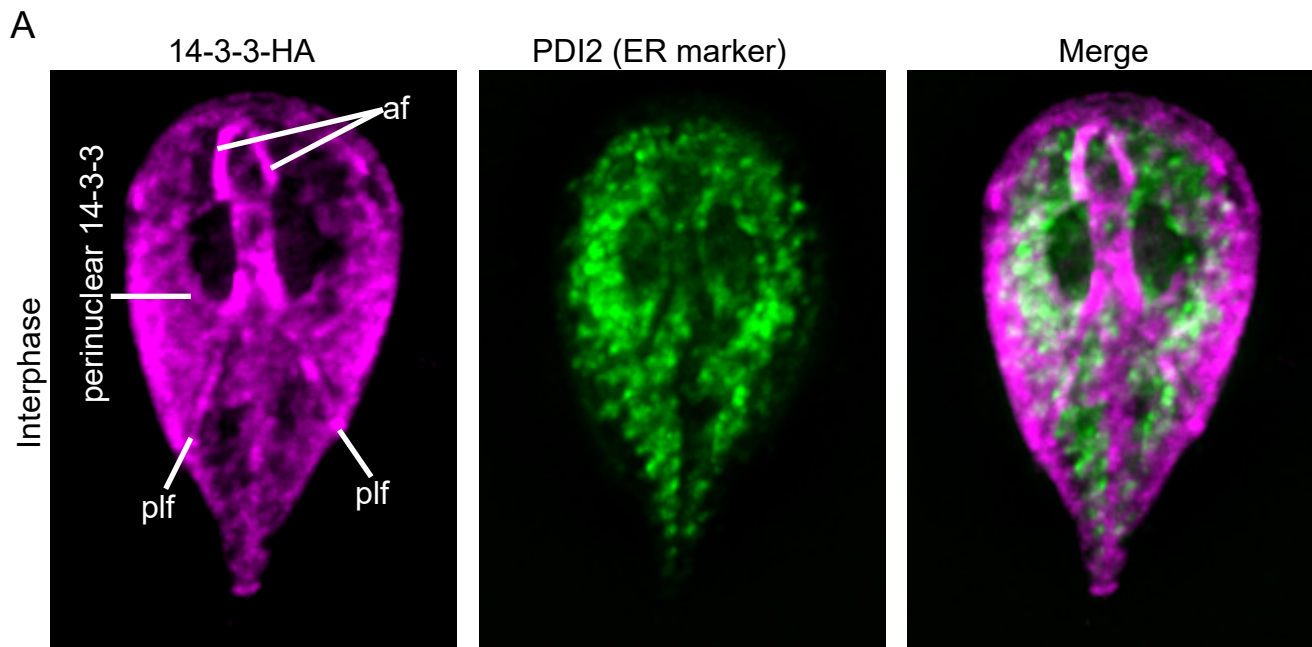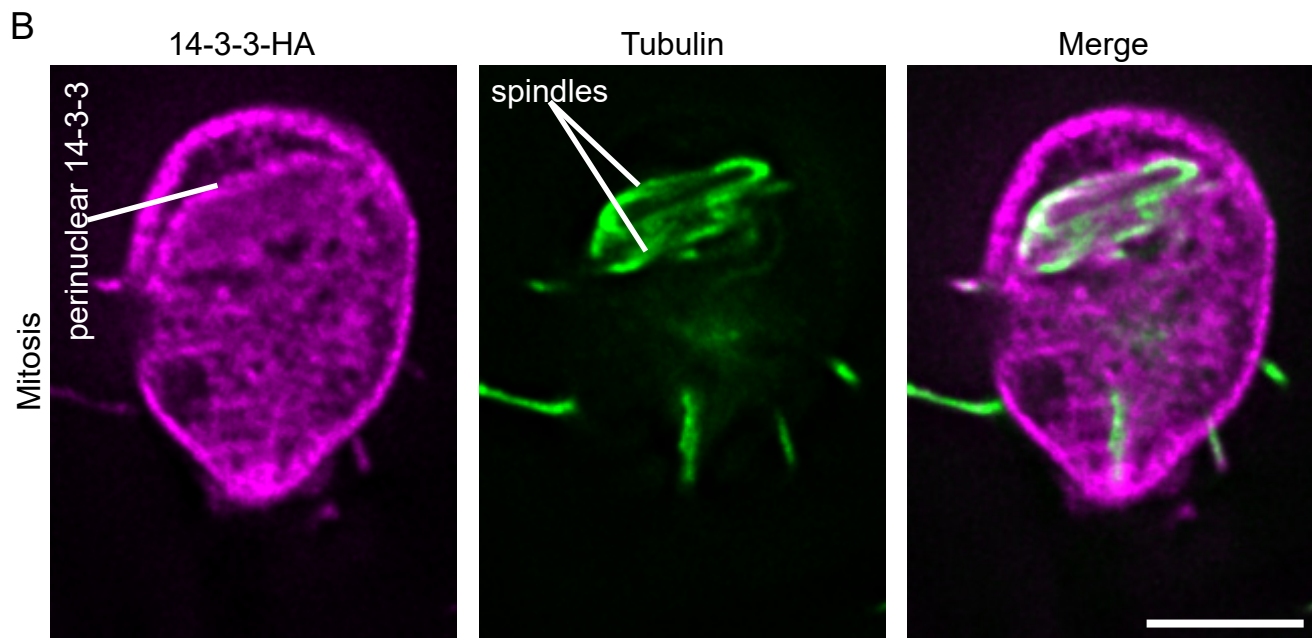

Supplement: FIG S2 [file sph005172359sf2.pdf]

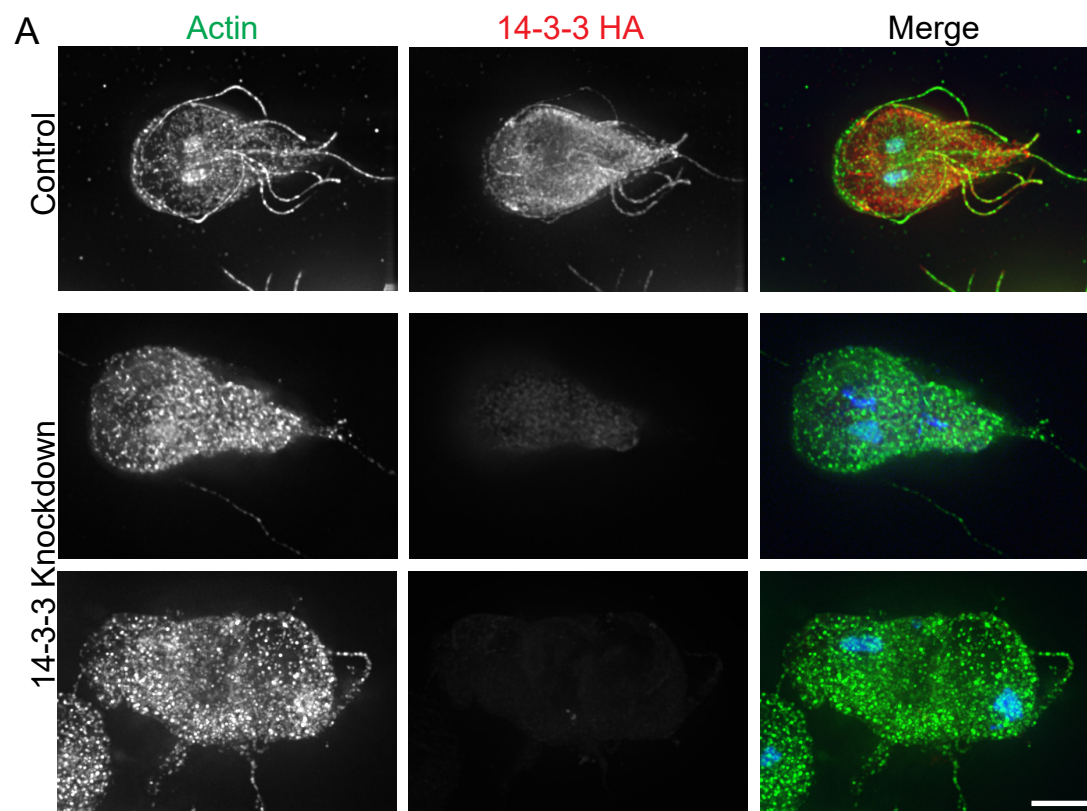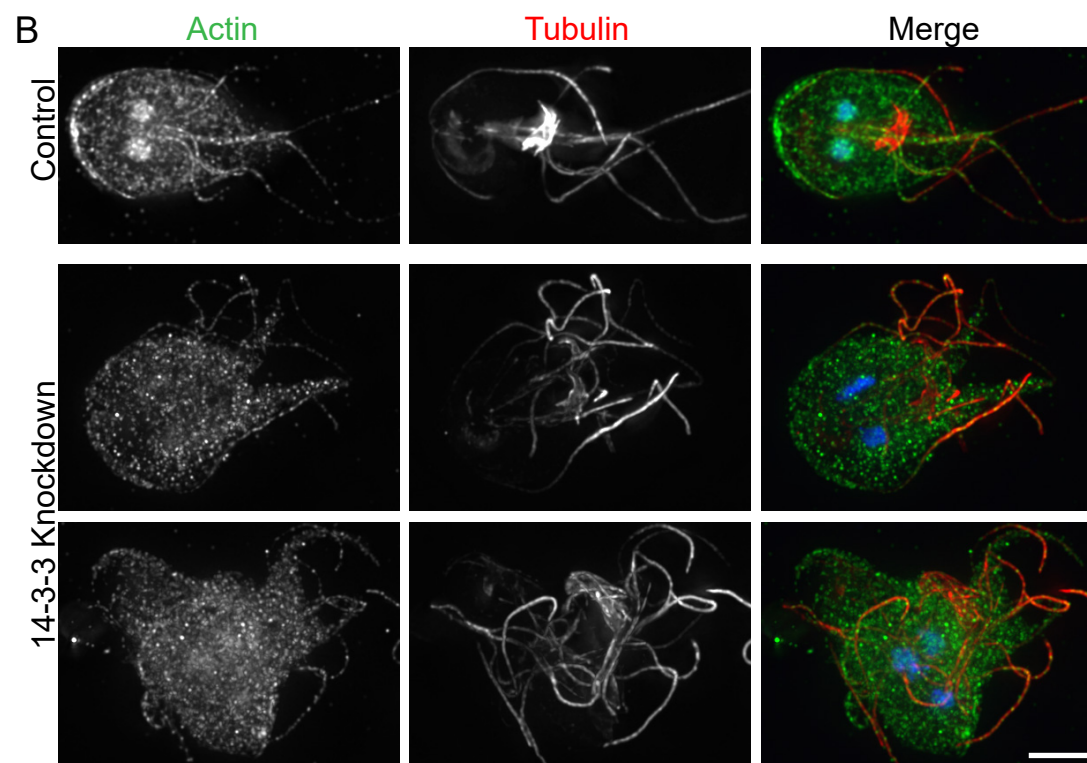

Supplement: FIG S3 [file sph005172359sf3.pdf]

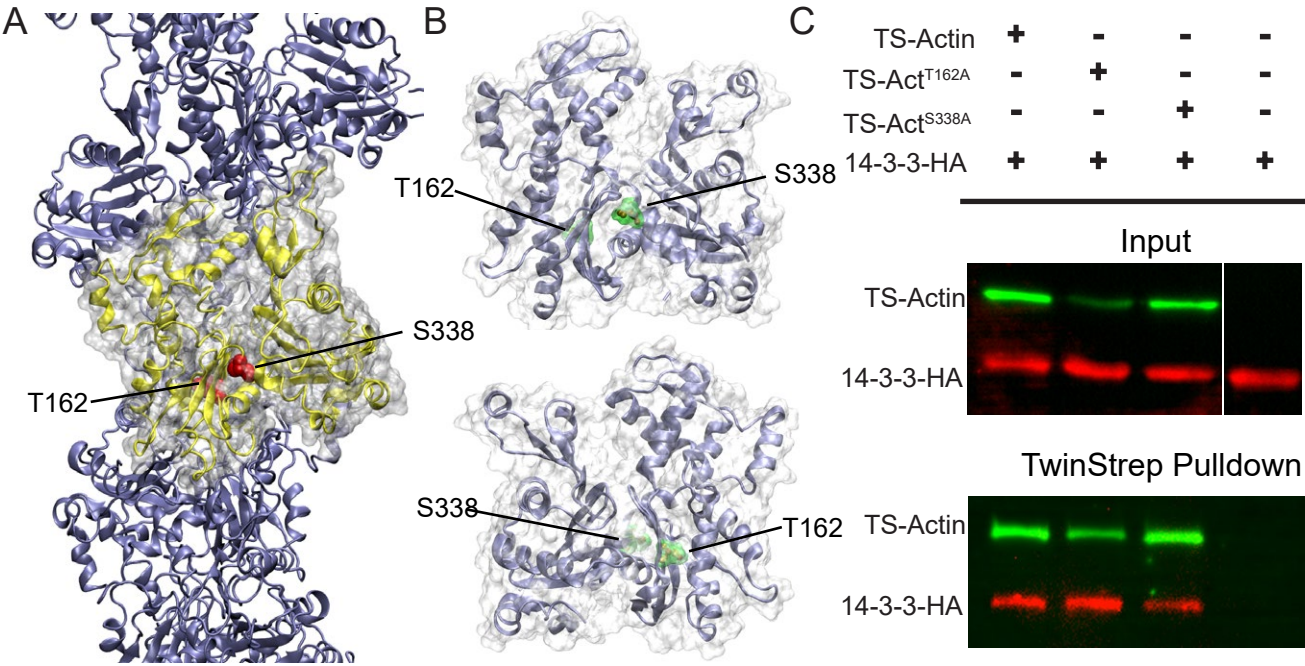

Supplement: FIG S4 [file sph005172359sf4.pdf]
