# Supplementary material for: 14-3-3 Regulates Actin Filament Formation in the Deep-Branching Eukaryote Giardia lamblia
Source: mSphere. 2017 Sep 13;2(5):e00248-17. doi: 10.1128/mSphere.00248-17 (PMC5597967; doi:10.1128/mSphere.00248-17)
Supplement: TABLE S1 [file sph005172359st5.docx]

**Table S1 Intersection between Gl-actin and Gl-14-3-3 interactome studies.**

| **IDs** | **Annotation** | **14-3-3 Peptides** | **Actin Peptides** | **kDa** |
| --- | --- | --- | --- | --- |
| GL50803_6430 | 14-3-3 | 21 | 8 | 28576 |
| GL50803_40817 | Actin | 3 | 1200 | 42000 |
| GL50803_16124 | TCP-1 chaperonin subunit eta | 13 | 196 | 64752 |
| GL50803_91919 | TCP-1 chaperonin subunit alpha | 10 | 82 | 59281 |
| GL50803_17411 | TCP-1 chaperonin sub gamma | 9 | 71 | 61559 |
| GL50803_11397 | TCP-1 chaperonin subunit beta | 9 | 204 | 56604 |
| GL50803_11992 | TCP-1 chaperonin sub epsilon | 8 | 223 | 61193 |
| GL50803_13500 | TCP-1 chaperonin subunit theta | 8 | 187 | 60646 |
| GL50803_17482 | TCP-1 chaperonin subunit alpha | 8 | 86 | 56324 |
| GL50803_10231 | TCP-1 chaperonin subunit zeta | 5 | 118 | 60941 |
| GL50803_9825 | TBP-interacting protein TIP49 | 6 | 276 | 51418 |
| GL50803_6886 | prokaryotic SMC domain protein | 11 | 11 | 102836 |
| GL50803_15251 | Hypothetical protein | 3 | 2 | 32493 |
| GL50803_15120 | Hypothetical protein | 1 | 4 | 26644 |
| GL50803_111950 | Axonmeal Dynein Heavy chain | 1 | 900 | 570319 |
| GL50803_42285 | Ciliary dynein heavy chain 11 | 1 | 48 | 834759 |
